# A Carbon Nanotube–Metal Oxide Hybrid Material for Visible-Blind Flexible UV-Sensor

**DOI:** 10.3390/mi11040368

**Published:** 2020-04-01

**Authors:** Pawan Pathak, Sanghoon Park, Hyoung Jin Cho

**Affiliations:** Department of Mechanical and Aerospace Engineering, University of Central Florida, Orlando, FL 32816, USA; sanghoon.park@ucf.edu

**Keywords:** ZnO, carbon nanotube (CNT), flexible sensor, photodetector

## Abstract

Flexible sensors with low fabrication cost, high sensitivity, and good stability are essential for the development of smart devices for wearable electronics, soft robotics, and electronic skins. Herein, we report a nanocomposite material based on carbon nanotube and metal oxide semiconductor for ultraviolet (UV) sensing applications, and its sensing behavior. The sensors were prepared by a screen-printing process under a low-temperature curing condition. The formation of a conducting string node and a sensing node could enhance a UV sensing response, which could be attributed to the uniform mixing of functionalized multi-walled carbon nanotubes and zinc oxide nanoparticles. A fabricated device has shown a fast response time of 1.2 s and a high recovery time of 0.8 s with good mechanical stability.

## 1. Introduction

The ozone layer has been depleted due to human activities, including the production of chlorofluorocarbon compounds, which has detrimentally disrupted the ecosystem resulting from the increased UV dose through the atmosphere [1,2]. The enhanced exposure to UV produces an adverse impact on human health and other living organisms [3]. Thus, UV sensors are important for monitoring UV radiation effectively and to avoid damage by excessive exposure. The UV sensors can also be used in other areas, for example, flame sensing, imaging, space communications, missile tracking, etc. [4,5,6]. It is imperative to monitor UV exposure levels in real time in order to prevent skin health-related risks such as inflammatory disorders, wrinkles, and skin cancer. The recent advancement of wearable electronics has generated a lot of attention and raised the demand for flexible sensors that can be integrated into existing technology. However, the commercially established UV sensors based on silicon technology are not suitable due to their mechanical rigidness and lack of UV selectiveness.

A nanomaterial-based metal oxide semiconductor has been recognized as an alternative to the silicon-based technology due to flexibility and the wide bandgap that can be exploited for UV sensing. Metal oxides can be assembled as a thin film on a wide range of substrates using a cost-effective wet chemical approach [7]. Among the various metal oxides, ZnO, CuO, and TiO_2_ are promising metal oxide semiconductors for their chemical stability, low toxicity, and high selectivity [8,9,10]. ZnO is one of the most widely studied oxide materials for UV sensing applications because of a high exciton binding energy of 60 meV and a wide bandgap of 3.37 eV [11,12]. However, pristine ZnO-based UV sensors have shown low sensitivity, which makes it unsuitable for the real-time measurement of UV radiation. Carbon is earth abundant, low cost material that can be introduced to a metal oxide semiconductor for an effective charge separation and transportation [13,14]. Various carbon allotropes such as fullerenes, carbon nanotubes, and graphene have been studied for nanoelectronics, optoelectronics, supercapacitors, and solar cell applications in the past few decades [15,16]. Several groups have studied the carbon nanotube metal oxide semiconductor network for UV sensing and gas sensing applications [17,18,19,20]. However, improvements in the performance of the device due to the incorporation of carbon allotropes is highly affected by its random aggregation [14]. Yi et al. have studied the influence of the presence of functional groups on the aggregation kinetics of the multiwalled carbon nanotube [21]. The result suggested that the oxidized carbon nanotube (carbonyl, carboxyl, and hydroxyl groups) is more stable to aggregation and deposition.

**Scheme 1:**
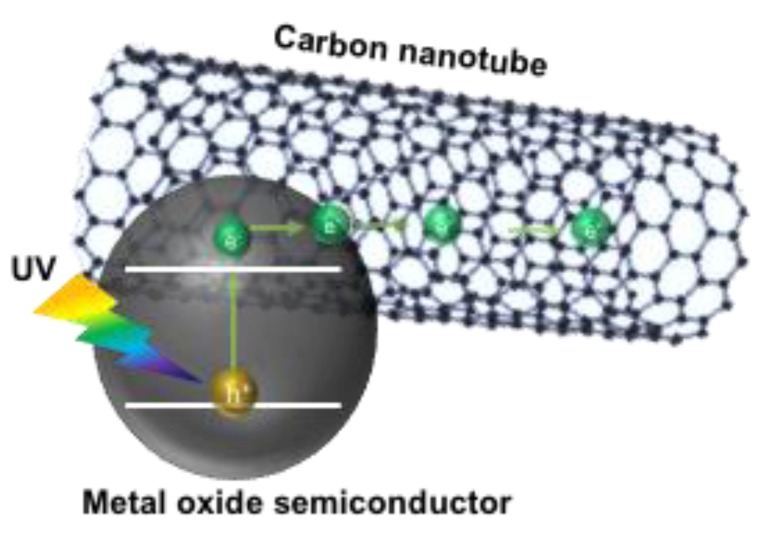
Schematic representation of the working principle of the nanocomposite sensor.

Scheme 1 presents the benefit of carbon nanotube in composite material. The presence of carbon nanotube can provide stable electrical connections between metal oxide semiconductors and planar interdigitated electrodes. Carbon nanotube collects and transmits free charge carriers without much loss, so the small change in the conductivity of metal oxide semiconductor particle could be reliably detected.

In this work, we have introduced a hybrid nanomaterial for flexible, visible-blind UV sensors. Both the electrode and sensing material were fabricated using a screen-printing method on a flexible polyethylene terephthalate (PET) substrate. The mixture of OH functionalized multi-walled carbon nanotubes (MWCNTs) (OH-MWCNT) and ZnO nanoparticles of different ratios was optimized with respect to the UV sensing performance. Also, a sensing mechanism of the device was studied using various characterization tools. It was found that the use of nanocomposite sensing material enhanced UV sensing characteristics, i.e., repeatability, response times, and mechanical stability. This strategy could be used to fabricate low cost, flexible, and wearable UV sensors.

## 2. Materials and Methods

### 2.1. Chemicals

Zinc acetate, ammonia solution, terpinol, ethanol, and methanol were obtained from Sigma Aldrich (St. Louis, MO, USA). Ethylene cellulose, silver paste, and multiwalled OH-MWCNT were obtained from Alfa Assar (Ward Hill, MA, USA), Daejoo Electronic Materials (Gyeonggi-Do, Korea), and Cheap Tubes (Cambridgeport, VT, USA), respectively. All these chemicals were used without additional treatment or purification.

### 2.2. ZnO Nanoparticle Synthesis

The approach used for the synthesis of ZnO nanoparticle is described elsewhere [22]. Briefly, 0.6 M zinc acetate solution was prepared in methanol solvent. The solution was stirred at 80 °C for two hours for well mixing. The pH of the solution was adjusted between 9 to 11 using an ammonia solution, and the solution was dried at 100 °C. Later, the temperature of the solution was further increased to 150 °C for the gelation process. The prepared ZnO nanoparticles were washed and dried several times to remove impurities. Finally, the ZnO nanoparticles were annealed at 500 °C for 2 hours to improve crystallinity.

### 2.3. Preparation of OH-MWCNT/ZnO Nanocomposite

The composite was formed by mixing ZnO and OH-MWCNT. In order to avoid the known dispersion problem with carbon nanotubes in a solvent, OH-functionalized MWCNTs were used to achieve uniform mixing and high interfacial bonding [23]. First, 2 g of ethylene cellulose was dissolved in 20 mL of ethanol and 78 mL of terpinol and stirred via a magnetic stirrer for 24 h. Ethylene cellulose was used to enhance the bonding strength between the sensing material and the substrate. Then different concentrations of the OH-MWCNT and ZnO were added to the mixture in such a way that the weight ratio of the solvent to the solution was 60:40.

### 2.4. Device Fabrication

**Figure 1:**
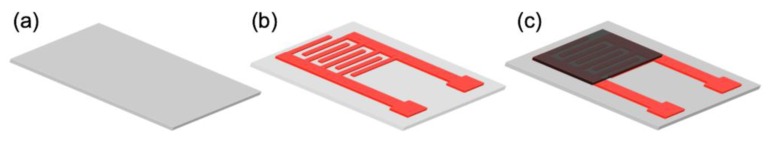
Schematic illustration of the fabrication of a flexible ultraviolet (UV) sensor; (**a**) polyethylene terephthalate (PET) substrate; (**b**) screen printed silver electrode; and (**c**) screen printed sensing layer.

Figure 1 shows the device fabrication procedure. Metal mesh screens (NBC Meshtec Americas Inc., Batavia, IL, USA) were used for printing inks for conducting and sensing layers. A PET substrate was used as a low cost and flexible substrate. First, silver interdigitated electrodes were printed manually by squeezing silver paste through the metal mesh screen to the PET substrate. After printing, samples were dried at room temperature for 4 h. Then, the OH-MWCNT/ZnO composite film was printed as a sensing layer on top of the planar interdigitated electrodes. The low-temperature treatment ensures that it would not thermally damage the PET substrate.

### 2.5. Materials Characterization

Electrical characterizations were performed using Keithley 2401 (Tektronix Inc., Beaverton, OR, USA) at room temperature. Optical characterization of the materials was carried out using an Agilent Cary 300 spectrophotometer (Agilent Technologies Inc. Santa Clara, CA, USA) in the wavelength range of 250−550 nm. The surface morphologies of the samples were probed using a Zeiss ultra 55 scanning electron microscope (SEM) system (Carl Zeiss SMT GmbH, Oberkochen, Germany) operated at 5 keV. The 365 nm centered laser was used as the laser source for UV sensing characterization. An X-ray photoelectron spectroscopy (XPS) were collected on ESCALAB™ XI+ X-ray Photoelectron Spectrometer Microprobe (Thermo Scientific, Waltham, MA, USA). Monochromatic, micro-focused Al Kα line was used to analyze the XPS of the sample.

## 3. Results and Discussion

### 3.1. Morphology Analysis

**Figure 2:**
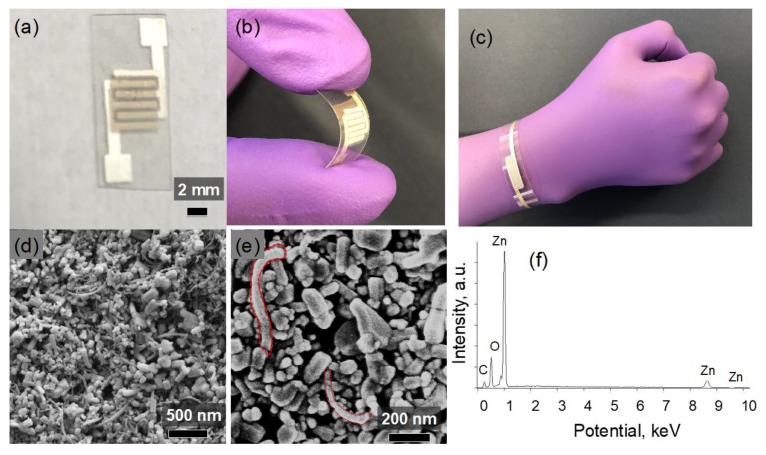
(**a**) Optical images of the fabricated sensor; (**b**,**c**) demonstrating the flexibility of the sensor; (**d**,**e**) scanning electron microscope (SEM) image of the sensing material at different magnification; and (**f**) elemental dispersive X-ray spectroscopy (EDX) of the sensing material showing the elemental composition.

Figure 2 shows the SEM image of the morphology of the composite material. The image reveals the tube-like structure of the OH-MWCNT and particle-like structure of ZnO. The OH-MWCNT was used as the conducting string and ZnO was used as the sensing node. The average carbon nanotube diameter is 15 nm with few microns in length, and the size of the average nanoparticles is 80 nm in diameter. Elemental dispersive X-ray spectroscopy (EDX) analysis shows the elemental composition of the sensing layer.

**Figure 3:**
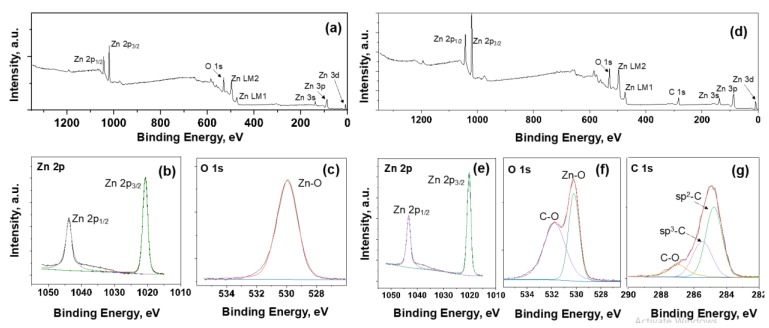
X-ray photoelectron spectroscopy (XPS) analysis of ZnO nanoparticles and OH functionalized multi-walled carbon nanotube (MWCNT)/ZnO composite. (**a**)Survey peak of ZnO; (**b**) Zn 2p spectra of ZnO; (**c**) O 1s spectra of ZnO; (**d**) Survey peak of composite; (**e**) Zn 2p spectra of composite; (**f**) O 1s spectra of composite; and (**g**) C 1s spectra of composite

The XPS spectra of ZnO and OH-MWCNT/ZnO is presented in Figure 3. The survey peak of the synthesized ZnO nanoparticles is shown in Figure 3a indicates the presence of zinc and oxygen. Figure 3b,c shows the high-resolution XPS spectra of Zn 2p and O 1S, respectively. The two clear, distinct peaks located at 1021.3 and 1044.4 eV are attributed to the spin-orbit of Zn 2p_3/2_ and Zn 2p_1/2,_ respectively. The energy splitting between the Zn 2p_1/2_ and Zn 2p_3/2_ is ~23.1 eV is in agreement with the reported value [24]. The O 1s core peak (Figure 3c) of ZnO nanoparticles shows one distinct peak at 529.8 eV associated with O^2-^ ions in the Zn–O bonding of the ZnO nanoparticle [24]. The survey peak of OH-MWCNT/ZnO composite is shown in Figure 3d indicates the presence of zinc, oxygen, and carbon. The high-resolution XPS peaks of the Zn presented in Figure 3e reveals two binding energy peaks at 1021.3 and 1044.4 eV associated with Zn 2p_1/2_ and Zn 2p_3/2_ spin-orbit splitting, respectively. The result indicates that there is no change in the chemical state of Zn in the OH-MWCNT/ZnO composite compared to that of Zn in the ZnO nanoparticle. However, the O 1s peak (Figure 3f) of OH-MWCNT/ZnO composite is resolved into two peaks centered at 529.8 and 532.0 eV, representing the Zn-O bonding and C–O, respectively. The C 1s peak (Figure 3g) was fitted into three peaks centered at 584.7, 585.4, and 586.9 eV. The peak centered at 586.9 eV is associated with C–O groups in the form of C–OH [25]. The sp^2^ and sp^3^ hybridized carbon peaks were observed at 284.7 and 285.4 eV respectively [26]. The result suggests the formation of a composite material.

### 3.2. Optical Absorbance of the Films

**Figure 4:**
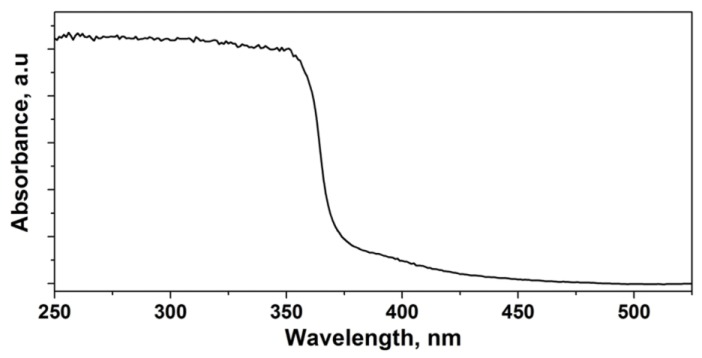
Room temperature optical absorbance spectra of the composite sensing material.

Figure 4 shows the absorbance spectra of composite material on a PET substrate from 250 nm to 550 nm. The absorption edge of the composite sensing material is 382 nm. It shows that the composite material can absorb UV radiation selectively without the need of optical filters.

### 3.3. Sensor Response

**Figure 5:**
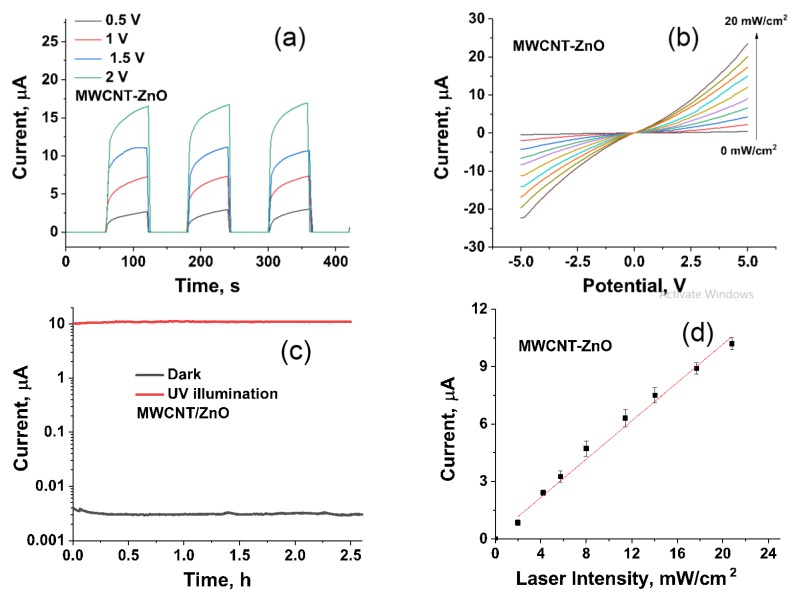

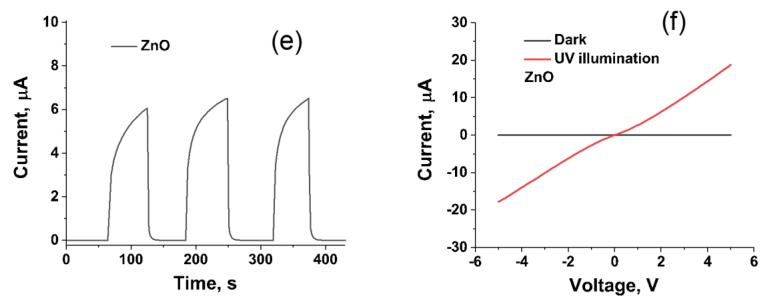
(**a**) Chronoamperometric responses of the OH-MWCNT/ZnO sensor towards cyclic exposure of 365 nm centered laser radiation at a bias voltage range of 0.5 V to 2 V; (**b**) current-voltage (I-V) characteristics of a fabricated nanocomposite sensor at a constant bias of 1.5 V measured in the range of 0 to 20 mW/cm^2^ UV illumination; (**c**) the time-dependent stability of the sensor over a duration of 2.5 h under dark and continuous illumination of 20 mW/cm^2^ UV radiation; (**d**) the variation of the current with the change in the illuminated UV intensity at the constant bias of 1.5 voltage; (**e**) chronoamperometric responses of a pristine ZnO sensor towards a cyclic exposure of 20 mW/cm^2^ UV illumination at a constant bias of 1.5; and (**f**) current-voltage characteristics of a fabricated ZnO sensor.

The devices (composite (OH-MWCNT/ZnO) and ZnO alone) have shown distinct electrical responses to the UV radiation, as shown in Figure 5. The current time characteristics of a fabricated nanocomposite sensor under cyclic on-off illumination of 20 mW/cm^2^ UV radiation at applied bias in the range of 0.5 V to 2 V is presented in Figure 5a. The response time and recovery time of the sensor calculated at 1.5 V bias current (Figure 5a) are 1.2 s and 0.8 s, respectively. The photocurrent increased with the increase in bias voltage. The current-voltage (I-V) characteristics of a fabricated nanocomposite sensor at a constant bias of 1.5 V measured in the range of 0 to 20 mW/ cm^2^ UV illumination conditions are shown in Figure 5b. As the intensity of UV radiation increased, the current response of the device increased. Figure 5c represents the time-dependent stability of the sensor over a duration of 2.5 h under a dark and continuous illumination of 20 mW/cm^2^ UV radiation. Figure 5c shows that electric conductivity increased by over three orders of magnitude upon exposure of UV radiation. In addition, the results show the stability of the sensor over prolonged exposure time. The variation of the current with the change in the illuminated UV intensity at the constant bias of 1.5 voltage is presented in Figure 5d. According to Figure 5d, the linear relation (R^2^ = 0.987) was observed between the photocurrent and the intensity of UV radiation. The result demonstrates the wide dynamic range of the fabricated device. The current time characteristics of a fabricated pristine ZnO sensor at a constant bias of 1.5 V measured under periodic exposure of 20 mW/cm^2^ UV radiation is shown in Figure 5e. The composite material has demonstrated a ~66% higher photocurrent response than ZnO alone (Figure 5a,e). The result shows the benefit of the composite material over the pristine metal oxide semiconductor. The current-voltage characteristics of a fabricated ZnO sensor measured under dark and 20 mW/ cm^2^ UV illuminated conditions are shown in Figure 5f.

**Figure 6:**
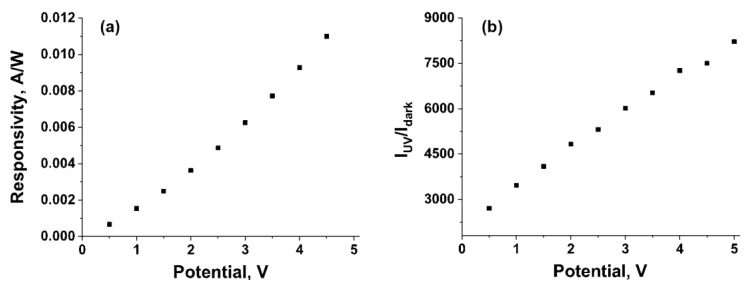
(**a**) Responsivity and (**b**) photoresponse (I_UV_/I_dark_) of the device under 365 nm laser radiation.

Responsivity measures the input-output gain of the sensor and is defined by the ratio of the photocurrent to the incident optical power of UV radiation [27]. Figure 6a shows the responsivity of the device with respect to change in the applied bias voltage. The responsivity increased with an increase in the bias voltage. The calculated value of the responsivity at an applied 5V bias was 0.011 A/W. The photoresponse (ratio of current at UV illumination to dark) with respect to applied bias is presented in Figure 6b. The data shows the nearly linear relation between the photoresponse and the applied bias.

The fabricated senor was compared with other previously reported sensors, as listed in Table 1. The result clearly shows the fabricated sensor had a higher photoresponse (I_UV_/I_dark_) value. The responsivity of the fabricated sensor could be further increased by increasing the amount of carbon nanotube content in the composite material, but decreased the photoresponse (I_UV_/I_dark_) due to a significant increase in the dark current.

### 3.4. Response of Device under Solar Radiation

**Figure 7:**
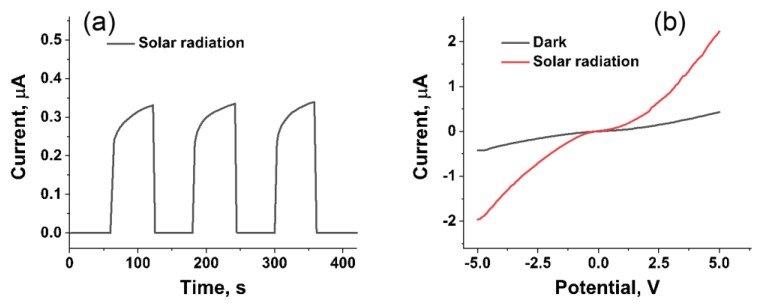
(**a**) Chronoamperometric response of the composite sensor towards cyclic exposure of solar radiation at a bias voltage of 1.5 V; and (**b**) IV characteristics of the device.

To demonstrate the real-life application of the device, the flexible fabricated composite sensor was tested under solar radiation. Figure 7a shows a cyclic UV response of the sensor, and Figure 7b shows an I-V characteristic of the device under solar radiation and the dark current radiation. This shows the potential application of the device as a real-time wearable UV sensor. The solar UV intensity was calculated using a linear equation obtained from Figure 5d was ~2.1 mW/cm^2^. The typical UV intensity of solar radiation (280 to 400 nm) is 2 to 5 mW/cm^2^ [28].

## 4. Conclusions

A flexible, wearable, and visible-blind nanocomposite UV sensor was fabricated on a PET substrate using a screen-printing method. The robust performance of the UV sensor was attributed to the collective and synergetic performance of carbon nanotube as a conductive node and the ZnO as a sensing node in the composite material. In addition, the use of functionalized carbon nanotube enhanced the interconnection between the carbon nanotube and metal oxide semiconductors. The photoresponses were observed under static and periodic UV radiation conditions, which demonstrated the reliability and repeatability of the proposed sensor. The fabricated sensor was also evaluated under solar UV radiation to demonstrate the applicability of the sensor for real-time monitoring of UV radiation for environmental safety and human healthcare.

## Figures and Tables

**Table 1:** Comparison of the fabricated sensor with the other reported sensors.

Material	Bias	Responsivity, A/W	I_UV_/I_dark_	Substrate	Ref.
Carbon/ZnO	1 V	0.38	<10	Rigid	[17]
MWCNT/ZnO	5 V	-	2.68	Rigid	[18]
CNT/ZnO	2 V	2.1	34	Rigid	[19]
MWCNT/ZnO	10 V	4.8 × 10^−4^	7.3 × 10^3^	Rigid	[20]
OH-MWCNT/ZnO	5 V	0.011	8.2 × 10^4^	Flexible	This work
